# A bioluminescent reporter bioassay for in-process assessment of chimeric antigen receptor lentiviral vector potency

**DOI:** 10.1093/abt/tbae032

**Published:** 2024-12-19

**Authors:** Julia K Gilden, Pete Stecha, Jim Hartnett, Mei Cong

**Affiliations:** Research and Development, Promega Corp., Madison, WI 53711, United States; Research and Development, Promega Corp., Madison, WI 53711, United States; Research and Development, Promega Corp., Madison, WI 53711, United States; Research and Development, Promega Corp., Madison, WI 53711, United States

**Keywords:** lentivirus, CAR-T, potency assay, bioassay

## Abstract

**Background:**

Chimeric antigen receptor (CAR)–T-cell therapy is a breakthrough in the field of cancer immunotherapy, wherein T cells are genetically modified to recognize and attack cancer cells. Delivery of the CAR gene is a critical step in this therapy and is usually achieved by transducing patient T cells with a lentiviral vector (LV). Because the LV is an essential component of CAR-T manufacturing, there is a need for simple bioassays that reflect the mechanism of action (MOA) of the LV and can measure LV potency with accuracy and specificity. Common methods for LV quantification may overestimate functional titer and lack a functional readout of LV MOA.

**Methods:**

We developed a bioluminescent reporter bioassay using Jurkat T cells stably expressing a luciferase reporter under the control of an nuclear factor of activated T cells (NFAT) response element and tested its suitability for measuring LV potency.

**Results:**

Jurkat reporter cells can be transduced with CAR LV and combined with target cells, yielding a luminescent signal that is dependent on the identity and potency of the LV used. Bioluminescence was highly correlated with CAR expression. The assay is stability indicating and suitable for use in drug development and quality control settings.

**Conclusions:**

We have developed a simple bioassay for potency testing of CAR LV. The bioassay represents a significant improvement over other approaches to LV quantification because it reflects the MOA of the LV and selectively detects fully functional viral particles, making it ideal for inclusion in a matrix of in-process quality control assays for CAR LV.

## Teaser

Statement of Significance: We have developed a simple and sensitive bioluminescent reporter bioassay for measuring chimeric antigen receptor–encoding lentiviral vector potency. This assay can be used for in-process quality control during the manufacturing of vectors for cell therapy.

## Introduction

Chimeric antigen receptor (CAR)-T cells are personalized living drugs that have shown remarkable efficacy against hematological malignancies. They are produced by *ex vivo* genetic modification of patient T cells to express a CAR that consists of an extracellular domain targeting a tumor-associated antigen (generally a single-chain variable fragment, or ScFv, derived from a monoclonal antibody) and one or more tandem intracellular signaling domains [1]. Upon binding of the CAR to an antigen-expressing target cell, immunoreceptor tyrosine–based activation motifs (ITAMSs) on the intracellular signaling domains are phosphorylated, leading to downstream signaling that ultimately leads the CAR-T cell to carry out effector functions such as cytotoxicity and cytokine production [2–4]. The design of the CAR, and especially the identity of the costimulatory signaling domains included, can affect the eventual fate and efficacy of the CAR-T cell. For instance, CARs containing 4-1BB signaling domains promote long-term persistence in vivo, while CD28 costimulatory domains yield CAR-T cells with a more short-lived effector phenotype [5]. Given the complexity of CAR-T products and their potential *in vivo* outcomes, it is imperative that quality control assays used during CAR-T development and manufacturing reflect the most important biological features of the product [6].

Multiple delivery methods for CAR genes are under active development, including nonviral methods such as mRNA and clustered regularly interspaced short palindromic repeats (CRISPR) gene editing, but lentiviral vector (LV)–mediated gene transfer is the primary technology currently being used in the clinic. Clinical efficacy depends on generating a CAR-T product with a high proportion of cells expressing the transgene, but safety concerns arise if the vector copy number is too high, underscoring the importance of reproducible manufacturing and accurate titration of CAR-encoding LV [7]. Regulators such as the US Food and Drug Administration recognize the importance of LV in CAR-T manufacturing and therefore require a complete drug substance package for the LV, including a potency bioassay that reflects the LV transgene mechanism of action (MOA) [8] Popular analytical methods for LV titration can overestimate transducing units (TUs) by orders of magnitude and correlate poorly with transduction efficiency. For instance, p24 enzyme-linked immunosorbent assay (ELISA) is an indirect method that does not distinguish functional, transduction-competent viral particles from defective particles lacking viral RNA, which leads to an overestimation of viral titers. Similarly, quantitation of viral RNA by qPCR does not guarantee that viral genomes are complete and packaged in transduction-competent particles, again leading to overestimation of LV potency. [9] Furthermore, because p24 ELISA and qPCR do not distinguish between transduction-competent and defective particles, they cannot be used to assess the stability of LV particles over time or under stress conditions. Flow cytometry analysis of transduced cells can be used to compute a functional titer but does not provide a functional readout of whether the transduced cells exhibit the desired biological activity and thus does not reflect the MOA of the transgene. While p24 ELISA, RNA quantification, and flow cytometry can provide useful information about LV preparations, we sought to develop a functional cell-based potency bioassay to complement those techniques and provide additional information about the capacity of a CAR-encoding LV to potentiate T-cell signaling.

Transduction of primary human T cells followed by measurement of antigen-stimulated IFN-γ secretion has previously been used in the study of LV potency and stability over time [10]. This method optimally represents the MOA of the CAR-encoding LV, but is time-consuming, labor-intensive, and suffers from the inherent variability of human donor material, making it unsuitable for quality control testing. The Jurkat cell line was derived from an acute T-cell leukemia and has been widely used in the study of T-cell signaling [11]. Jurkat cells can be engineered to stably express bioluminescent reporters that respond to activating and inhibitory signals through the T-cell receptor, costimulatory and coinhibitory checkpoint receptors, and cytokine receptors [12, 13]. Bioluminescent reporters are ideal for these assays because of their high sensitivity and dynamic range relative to colorimetric and fluorescent reporters. Bioluminescent Jurkat reporter cells can be easily cloned and scaled to produce large cell banks for optimal reproducibility, making them established tools in the development and potency testing of monoclonal antibody drugs. Furthermore, because Jurkat reporter cells recapitulate much of the signaling downstream of CARs, they have been employed for screening and characterization of novel T Cell Receptors (TCRs), ScFvs, and CAR constructs [14–18]. We hypothesized that Jurkat reporter cell technology could be effectively adapted to allow for in-process MOA-reflective potency testing of lentiviral particles for CAR-T manufacturing. Unlike methods such as p24 ELISA, which may over or underestimate viral titers, this assay provides a functional readout directly linked to the mechanism of action, offering a more accurate reflection of lentiviral vector potency.

## Materials and methods

### Cell lines

Jurkat/NFAT-Luc2 reporter cells were generated by electroporation of Jurkat clone E6-1 (American Type Culture Collection) using the Lonza Nucleofector system. Transfected cells were enriched by hygromycin (Gibco) selection and cloned by limiting dilution. A clone was selected based on functional activity and passage stability. Raji cells were purchased from ATCC. For CRISPR knockout of CD19 in Raji cells, crRNA and tracrRNA (Integrated DNA Technologies) were incubated for 5 min at 95°C and cooled to room temperature. Then, Cas9 protein (IDT) was added and incubated for 20 min at room temperature to yield the ribonucleoproteins, which were then transfected into the cells. Single clones were sorted and screened based on loss of CD19 expression by flow cytometry. Knockout was confirmed by sequencing of the CD19 locus. Raji and Jurkat cells were maintained in RPMI media (Gibco) supplemented with 10% fetal bovine serum (Seradigm).

### Vectors and lentivirus particles

The NFAT-Luc2 reporter plasmid and CD19 and CD20 CAR expression plasmids were generated at Promega Corp. LV particles were generated by VectorBuilder. The CD19-CAR consisted of an anti-CD19 ScFv [19] followed by the CD8 transmembrane and hinge regions, then 4-1BB and CD3z intracellular signaling domains.

### Reporter bioassay

Jurkat/NFAT-Luc2 reporter cells were plated at a density of 20 000 cells per well of a white U-bottom 96-well plate (Costar) in 25 μl. Next, 25 μl diluted LV was added, and plates were centrifuged at 800×G for 30 min at 32°C. Cells were held in a 37°C 5% CO_2_ incubator for 4–24 h before the addition of 25 μl assay media (RPMI with 10% heat-inactivated FBS) per well. After an additional 24 h, cells were pelleted, and media were removed from the assay plates. Immediately, 50 μl fresh assay media, along with 50 μl Raji target cells (5000 cells per well or as indicated) were added to the assay wells. Each well was then gently mixed using a multichannel pipet. After 6 h of coculture in a 37°C 5% CO_2_ incubator, plates were equilibrated to room temperature, and 1 volume (100 μl) of Bio-Glo™ Reagent (Promega Corp) was added to each well. Luminescence was read on a GloMax® Discover plate-reading luminometer. Data were analyzed for four-parameter logistic (4PL) fit using GraphPad Prism 10 and JMP17® software.

### Flow cytometry

Cells were washed in PBS with 1% FBS and then stained with CD19 CAR Detection Reagent and Anti-biotin-PE (Miltenyi) according to the manufacturer’s instructions. Data were collected on a BD Accuri™ C6 Plus flow cytometer (BD Biosciences) and analyzed using Flowjo version 10 (FlowJo LLC).

### Antibodies and reagents

αCD3 antibody (clone OKT3) was obtained from Biolegend. Goat-α-mouse crosslinking antibody was from Jackson ImmunoResearch Labs. Blinatumamab (Blincyto™) was obtained from Myonex.

## Results

### Jurkat/NFAT reporter cells

**Figure 1:**
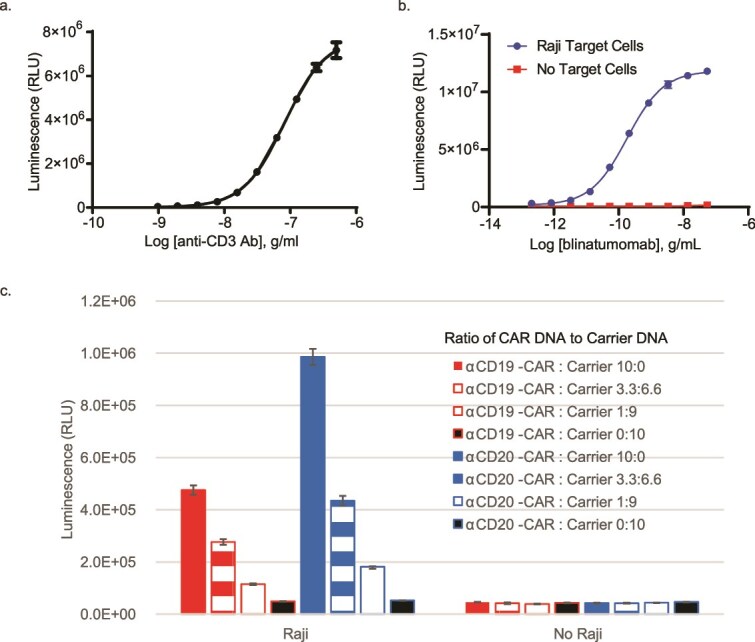
NFAT-Luc2 activity stimulated by multiple T-cell activation signals. (a) Jurkat/NFAT-Luc2 cells were incubated with α-CD3 antibody crosslinked with goat α-mouse IgG antibody at the indicated concentrations. (b) Jurkat/NFAT-Luc2 cells were incubated with or without Raji target cells and the indicated concentrations of blinatumomab. The curves were compared using an extra-sum-of-squares *F* test and were determined to be different (*P* > .0001) (c) Jurkat/NFAT-Luc2 cells were transiently transfected with varying concentrations of α-CD19-CAR or α-CD20-CAR plasmid DNA, balanced with carrier DNA (pGEM-3z) such that all conditions received the same total amount of DNA. One day after transfection, cells were incubated with or without Raji target cells. In all panels, assays were incubated for 6 h at 37°C, and luciferase activity was detected using the Bio-Glo™ Luciferase Assay System. Data are representative of at least three independent experiments with *n* = 3 technical replicates. Error bars indicate standard deviation. For (c), a one-way ANOVA was performed separately for a-CD19 and a-CD20 conditions. Dunnett’s multiple comparison post-testing found a significant difference between the carrier DNA only (0:1) condition and the CAR DNA conditions in the presence of Raji cells (*P* < .0001) and no differences in the absence of Raji cells. Post-testing for linear trend found a relationship between CAR DNA quantity and assay response in the presence of Raji cells (*P* < .0001).

All CARs currently being used in the clinic contain a CD3ζ domain. When the CAR is engaged by an antigen-expressing target cell, a signaling complex is recruited that ultimately results in the translocation of NFAT to the nucleus and transcription of activation-related genes. We have developed a Jurkat reporter cell line that stably expresses firefly luciferase under the control of an NFAT response element. Figure 1 demonstrates that these cells produced a bright luciferase signal directly proportional to the extent of T-cell receptor ligation. Crosslinking of the T-cell receptor can be achieved by antibody crosslinking (Fig. 1a) or using a bispecific antibody that recognizes a tumor-associated antigen in a coculture system (Fig. 1b). We hypothesized that the expression of a CAR and coculture could also induce signaling. To this end, we transiently transfected the reporter cells with a CD20- or CD19-specific CAR and combined them with Raji B cells, which express both antigens. Luciferase activity was directly correlated with the amount of CAR DNA used and was dependent on the presence of antigen-bearing target cells (Fig. 1c.)

### Luciferase reporter activity was titratable and specific

**Figure 2:**
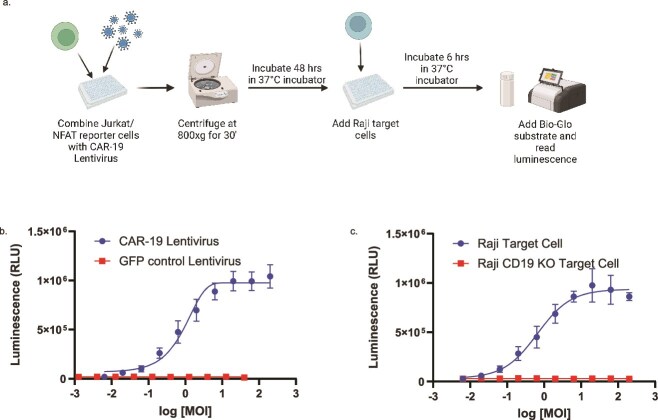
NFAT-Luc2 activity induced by CAR-19 LV and target cell coculture. (a) Schematic representation of assay workflow. (b) Jurkat/NFAT-Luc2 cells were transduced with CAR-19 or GFP control LV at the indicated MOI and incubated with wild-type Raji target cells. Only CAR-19 LV transduction led to a dose-dependent luminescent signal. (c) Jurkat/NFAT-Luc2 cells were transduced with CAR-19 LV and incubated with wild-type or CD19-knockout Raji target cells. A luminescent response was observed only with target cells expressing CD19. The curves within each panel were compared using extra-sum-of-squares *F* tests and were determined in each case to be different (*P* > .0001). Data are representative of at least two independent experiments with *n* = 3 technical replicates. Error bars indicate standard deviation. MOI = multiplicity of infection.

Next, it was tested whether the transduction of Jurkat/NFAT reporter cells with CAR lentivirus resulted in a titratable luminescent signal. To this end, the reporter cells were transduced with CAR-19 LV, allowed 48 h for full CAR expression, and subsequently cocultured with Raji target cells, resulting in a sigmoidal dose–response curve (Fig. 2a). By contrast, reporter cells transduced with a GFP-encoding LV showed no dose-dependent response when cocultured with Raji cells, demonstrating that the luciferase signal observed is not a nonspecific consequence of viral transduction or coculture (Fig. 2b). To further establish the specificity of the system, we transduced cells with CAR-19 LV and then cocultured them with wild-type Raji cells or CD19 knockout Raji cells. As demonstrated in Fig. 2c, effector cells exhibited an LV-dependent luciferase reporter response only when the target antigen was expressed.

### Luciferase activity correlates with chimeric antigen receptor expression

**Figure 3:**
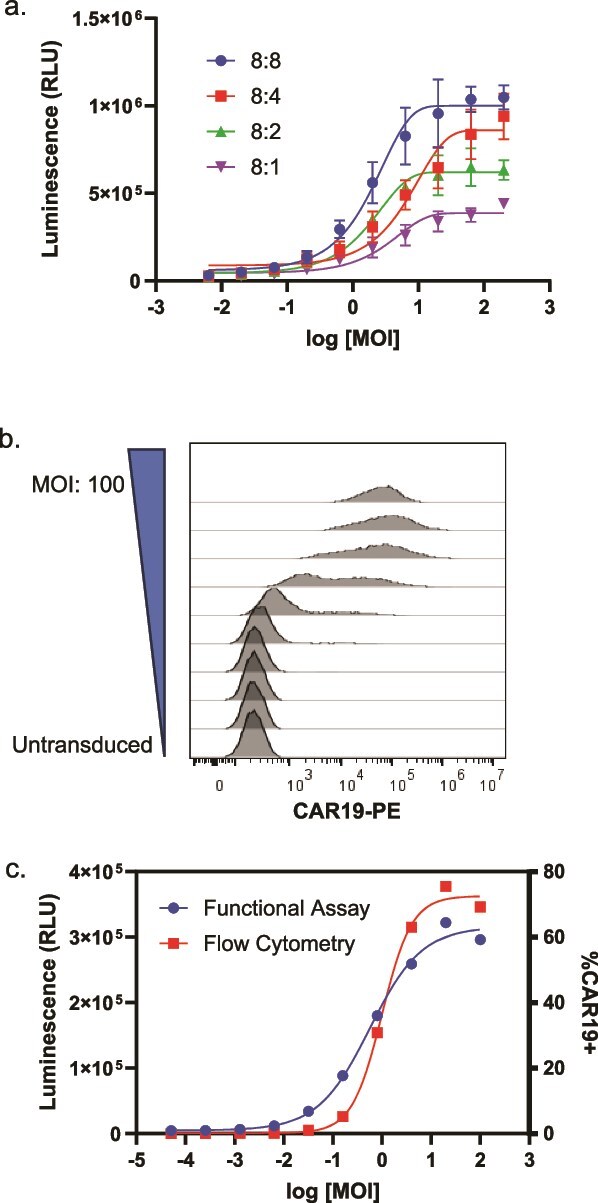
Correlation between flow cytometry and functional bioassay. (a) Jurkat/NFAT-Luc2 cells were transduced with CAR-19 LV and then combined with Raji target cells at the indicated E:T ratios. Luminescence was measured after a 6-h incubation at 37°C. The upper asymptotes and EC50s of the curves were compared using extra-sum-of-squares *F* tests, which indicated differences among the curves (*P* < .0001). *Post hoc* testing revealed a weak linear relationship between the E:T ratio and upper asymptote (*R*^2 = 0.56) and no relationship between E:T ratio and IC50 (*R*^2 = 0.03, slope not significantly different from 0; data not shown.) (b) Jurkat/NFAT-Luc2 cells were transduced with CAR-19 LV at the indicated MOI and stained for flow cytometry to measure surface expression of α-CD19 CAR. (c) Overlay of flow cytometry and functional bioassay data generated in parallel in a single experiment. Error bars indicate a standard deviation of *n* = 3 replicates.

Under the conditions used, we observed saturated bioluminescence at a multiplicity of infection (MOI) of ~10, and we sought to determine what factor(s) account for this saturation. First, we were able to rule out that the assay was reaching the limit of the luminometer, saturating substrate availability, or saturated NFAT occupancy based on the higher dose-dependent luminescence observed with blinatumomab and Raji target cells (Fig. 1b). Next, we evaluated whether at high MOI, the number of expressed CAR might exceed the number of potential target binding sites on the Raji target cells. To this end, we performed the assay with a fixed number of Jurkat reporter effector cells and varied the target cell density, such that the effector-to-target (E:T) ratio ranged from 8:8 to 8:1. While the maximum assay response changed as a function of target cell number, we did not observe a relationship between the target cell number and EC50 (Fig. 3a). This indicates that, within the range of E:T ratios tested, target cell availability does not impact the MOI at which the assay reaches its maximum response.

Finally, we tested whether the upper asymptote may be reached due to the saturated transduction of permissive effector cells. To evaluate this, we transduced reporter cells with CAR-19 LV and performed flow cytometry for CAR expression in parallel with the reporter bioassay. We found that the maximum bioluminescent signal corresponded with both the maximum proportion of cells expressing CD19-CAR and the maximum CAR-19 expression on a per-cell basis (Fig. 3b and c). This supports the hypothesis that at an MOI of 10, the assay reaches an upper asymptote because all permissive effector cells have been fully transduced with the CAR lentivirus.

### Assay precision and accuracy

**Figure 4:**
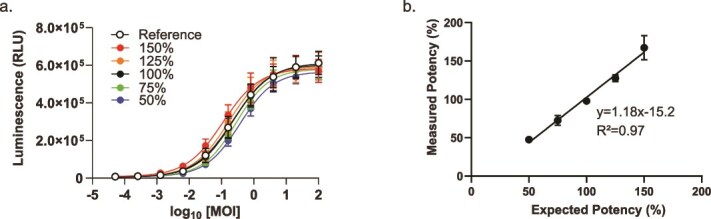
Prequalification of LV potency assay. Mock potency samples were prepared with relative potencies between 50% and 150% of the reference standard and then used in the LV potency assay. JMP17® software was used to fit 4PL curves, test for parallelism (*F* test), and calculate the measured relative potency of each sample. (a) Representative data from one of three independent experiments. Error bars indicate a standard deviation of *n* = 3 replicates. (b) Analysis of assay linearity based on pooled average recovery from all three experiments.

Assays intended for use in the quality control of commercial drug substances must have sufficient accuracy, precision, and linearity to support a potency specification [20]. Accurate and precise assays are essential to consistently determine a drug’s therapeutic effect at a given dose. Inaccurate or imprecise assays can increase the risk of treatment failure and adverse events. We evaluated these properties of the LV potency assay by preparing mock potency samples between 50% and 150% of a reference control sample. Three independent experiments were performed. Figure 4 shows a sample result of this study. With all three repetitions taken together, the accuracy (average percent recovery) was 99.4%, and the intermediate precision was 5% (Table 1). By plotting the expected and observed recovery values for each sample, we determined that the assay was linear with a slope of 1.18 and an *R*^2^ value of 0.97 (Fig. 4).

### Stability

**Figure 5:**
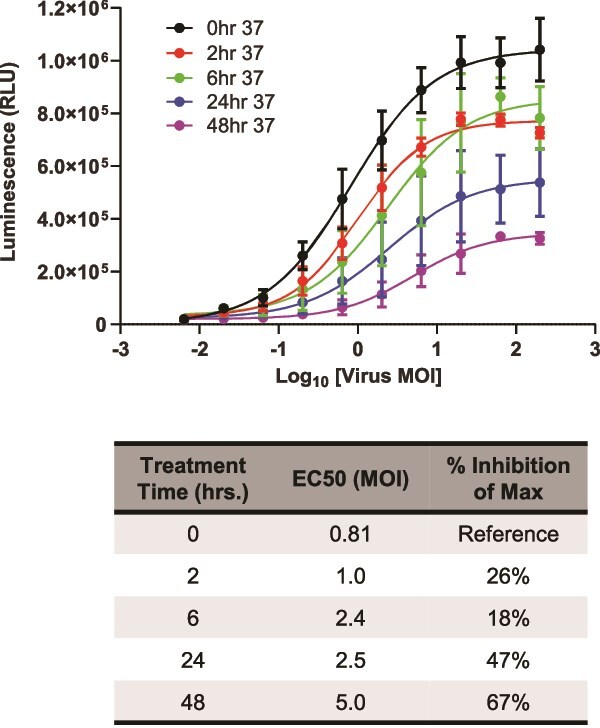
LV potency assay is stability indicating. CAR-19 LV was incubated at 37°C for the indicated times and then used to transduce Jurkat/NFAT-Luc2 cells, which were then incubated with Raji target cells for 6 h at 37°C. Luciferase activity was detected using the Bio-Glo™ Luciferase Assay System. Data were analyzed for 4PL fit, and the upper asymptotes and EC50s of the curves were compared using extra-sum-of-squares *F* tests, which indicated differences among the curves (*P* < .0001). *F* tests did not detect differences between the hill slopes or lower asymptotes.

Finally, guidelines require that potency assays for lot release must indicate the stability of the drug substance [20]. To evaluate whether our assay could distinguish active and degraded LV particles, we created forced degradation samples by incubating aliquots of LV at 37°C for up to 4 days prior to performing the assay. We observed that as incubation time increased, the dose–response curves shifted to the right, indicating decreased LV potency (Fig. 5). In addition, using heat-treated LV in the assay led to lower maximum assay responses, further demonstrating that assay results are affected by degraded LV.

## Discussion

In-process MOA-based potency testing of drug substances such as LV particles is required for regulatory approval of drug products derived from those drug substances, such as CAR-T cells [8]. In this study, we tested whether transduction of Jurkat reporter cells could be a good approach to potency testing of CAR LV. We found that reporter activity was dependent on both the expression of an appropriate CAR and on coculture with a target cell expressing the antigen of interest. Furthermore, we found that reporter activity correlated directly with CAR expression, regardless of whether cells were electroporated with a CAR expression plasmid or transduced with CAR LV.

Here we demonstrated that the Jurkat/NFAT reporter bioassay provides a precise and accurate method of quantifying CAR LV potency. A prequalification experiment using mock potency samples of CAR-19 LV showed high precision, as represented by the low average %CV over the course of three independent experiments, as well as relative potency measurements that closely matched the expected potencies across the tested range of 50%–150%. Interestingly, when we generated forced degradation samples by heat-stressing lentiviral particles, we found that both EC50 and maximum assay response were affected. This may reflect that stressed LV particles can still bind to Jurkat cells but not mediate successful transduction, thus blocking the binding of the remaining functional particles in the sample and reducing the maximum potential transduction efficiency. Taken together, the data demonstrate that this assay can be used in a matrix approach with analytical assays to more fully characterize the activity of LV.

Under ideally controlled assay conditions, LV titer would be the only factor influencing NFAT reporter activity. However, NFAT can integrate a wide variety of calcium-mediated signals in addition to antigen receptor signaling and may be sensitive to factors such as impurities and excipients in the LV prep, batch-to-batch variation in fetal bovine serum (FBS), metabolic state of the reporter cell, and expression of activating or inhibitory coreceptors on the target cell [21]. In this study, the use of antigen-knockout target cells and CAR-negative LV increases the confidence that the reporter activity we observed was due to antigen-dependent CAR signaling by transduced cells. We also mitigated the risk of some confounding variables by using thaw-and-use reporter cells, which can reduce assay variation that may otherwise arise due to inconsistent culture conditions. Still, as with any potency assay, suitable reference material must be identified for accurate relative potency determination [6]. This assay should not be used to measure the relative potency of LV particles carrying different CARs or using different vector backbones, as changes to the vector would obscure whether differences in reporter activity were due to differences in TU concentration or in the function of the CAR.

While currently approved CAR-T therapies are built by transduction of a single chimeric receptor into cells, many next-generation CAR-T products under development have multiple genetic modifications and more multifaceted MOAs [22]. These products include logic-gated CARs that restrict CAR expression of function to the tumor microenvironment as well as “armored CARs” that seek to modulate the tumor microenvironment by secreting soluble factors that cause inflammation, recruit particular immune cell populations, or suppress effector functions that might lead to dose-limiting toxicities [23–25]. As development progresses for these ever more complex products, in-process assays that test the potency of a wide variety of gene-modifying components will be critical. Based on our results with the NFAT reporter and CD19 CAR, it is likely that bioluminescent reporter cells can be exploited to develop MOA-reflective potency bioassays for those other delivered genes as well. This could include systems like the one described, where a signal is directly dependent on the transduced cells, or potentially a system where the transduced cell produces a soluble or membrane-bound factor that induces reporter signaling on a second cell population. For instance, a cytokine expression vector could be potency tested by monitoring reporter activity in a cytokine receptor–expressing target cell cocultured with the transduced Jurkat effector. We have recently demonstrated that TCRαβ-knockout Jurkat reporter cells are advantageous for the characterization of transgenic TCRs because they lack the mismatched pairing of endogenous and transgenic TCRα and β chains that occurs in parental Jurkat cells [26]. Delivery of tumor-specific TCRs to primary T cells, known as TCR-T therapy, is a particularly promising approach for solid tumors and intracellular tumor antigens. TCRαβ-knockout Jurkat reporter cells would be an ideal system for potency testing of TCR-carrying therapeutic LV.

**Table 1:** Summary of assay prequalification as described in Fig. 4.

Expected relative potency	Measured relative potency	Accuracy (% Recovery)	Precision (%CV)
50	46.6	93%	4%
75	73.1	97%	6%
100	97.8	98%	1%
125	128.1	102%	3%
150	160.3	107%	10%
Intermediate precision			5%

Reporter cells can also be utilized in potency testing of vectors in non-CAR gene therapies. While the Jurkat/NFAT system is an “off-the-shelf” reporter cell that can likely reflect the activity of any standard CAR lentivirus, some reporter systems may need to be purpose-built for specific cell and gene therapy programs. For instance, Pavlou *et al*. created a reporter bioassay to measure the potency of an Adeno-Assoviated Virus (AAV) encoding the CNGA3 calcium channel by using a genetically encoded calcium indicator that fluoresces when bound to calcium [27]. Development and characterization of reporter cell lines for key signaling pathways that can act as building blocks for gene therapy potency assays has the potential to accelerate the development of future gene therapies.

## Conclusion

We have demonstrated that Jurkat NFAT-luciferase reporter cells can be used as a simple MOA-reflecting bioassay for in-process potency testing of CAR-carrying LV. When LV was transduced into these cells, a dose-dependent luciferase response was observed, and this signal was wholly dependent on CAR recognition of a specific antigen on the surface of a target cell. This assay addresses shortcomings of current methods of LV quantification, such as p24 ELISA and qPCR, which do not distinguish complete, transduction-capable particles from nonfunctional or damaged material. The assay represents an improvement in both workflow and mechanistic relevancy when compared with flow cytometry assays that measure only the expression of CAR on the T-cell surface, making it ideal for inclusion in a matrix of quality control assays for LV drug substances.

## Data Availability

The data that support the findings of this study are available from the first author, J.K.G., upon reasonable request.
